# Supplementary material for: Intakes of Total, Free, and Naturally Occurring Sugars in the French-Speaking Adult Population of the Province of Québec, Canada: The PREDISE Study
Source: Nutrients. 2019 Sep 30;11(10):2317. doi: 10.3390/nu11102317 (PMC6835783; doi:10.3390/nu11102317)
Supplement: Supplementary file 1 [file nutrients-11-02317-s001.pdf]

Table S1 : Unadjusted mean (95% CI) intakes of total, free and naturally occurring sugars, expressed in grams, percentage of total sugars and percentage of energy they provide, in a representative sample of French-speaking adults of the Province of Québec, Canada

			Percentage of total sugars, %			Energy provided by, %kcal		
	n (weighted)	Total sugars, g	Free sugars	Naturally occurring sugars	Energy, kcal	Total sugars	Free sugars	Naturally occurring sugars
Sex								
Women	576	104.9 (101.4-108.4)^{a}	57.0 (55.7-58.4)^{a}	43.0 (41.6-44.3)^{a}	2116 (2071-2161)^{a}	19.8 (19.3-20.3)	11.5 (11.0-11.9)	8.3 (8.0-8.7)^{a}
Men	571	128.1 (122.9-133.3)^{b}	62.2 (60.8-63.6)^{b}	37.8 (36.4-39.2)^{b}	2692 (2624-2759)^{b}	18.8 (18.2-19.3)	12.0 (11.5-12.5)	6.7 (6.4-7.0)^{b}
P		<0.0001	<0.0001	<0.0001	<0.0001	0.03	0.03	<0.0001
Age group, y								
18-34	408	121.7 (116.0-127.4)^{a}	62.5 (60.9-64.1)^{a}	37.5 (35.9-39.1)^{a}	2461 (2386-2536)^{a}	19.6 (19.0-20.2)	12.5 (12.0-13.1)^{a}	7.1 (6.7-7.5)
35-49	338	116.6 (110.8-122.3)^{b}	58.6 (56.7-60.5)^{b}	41.4 (39.5-43.3)^{b}	2432 (2364-2500)^{b}	19.0 (18.3-19.7)	11.5 (10.8-12.2)^{b}	7.5 (7.1-7.9)
50-65	400	111.0 (106.1-115.8)^{c}	57.4 (55.8-59.1)^{b}	42.6 (40.9-44.2)^{b}	2317 (2253-2382)^{c}	19.2 (18.6-19.7)	11.2 (10.7-11.7)^{b}	8.0 (7.6-8.3)
P		<0.0001	<0.0001	<0.0001	<0.0001	0.33	0.0003	0.001
Administrative region								
Estrie	110	111.0 (101.3-120.6)	59.0 (55.6-62.3)	41.0 (37.7-44.4)	2390 (2288-2493)	18.3 (17.2-19.4)	11.1 (10.0-12.1)	7.2 (6.5-7.9)
Saguenay-Lac-Saint-Jean	107	129.8 (118.9-140.6)	63.9 (61.0-66.8)	36.1 (33.2-39.0)	2510(2372-2647)	20.6 (19.4-21.7)	13.4 (12.3-14.5)	7.2 (6.6-7.8)
Capitale-Nationale/ Chaudière-Appalaches	435	116.6 (111.2-121.9)	59.3 (57.6-60.9)	40.7 (39.1-42.4)	2408 (2340-24745)	19.2 (18.6-19.8)	11.6 (11.1-12.2)	7.6 (7.2-7.9)
Montréal	397	114.7 (109.7-119.8)	58.3 (56.6-59.9)	41.7 (40.1-43.4)	2375(2306-2443)	19.3 (18.7-19.9)	11.5 (11.0-12.0)	7.8 (7.4-8.2)
Mauricie	99	114.4 (103.2-125.6)	62.4 (59.3-65.4)	37.6 (34.6-40.7)	23889(2243-2534)	19.2 (17.8-20.5)	12.1 (11.1-13.2)	7.0 (6.2-7.8)
P		0.17	0.06	0.06	0.85	0.07	0.03	0.49
BMI group, kg/m^2								
Normal (< 25.0)	453	117.8 (112.9-122.6)^{a}	59.0 (57.4-60.5)	41.0 (39.5-42.6)	2346 (2284-2409)^{a}	19.9 (19.4-20.4)	12.0 (11.5-12.5)	7.9 (7.6-8.3)
Overweight (25.0-29.9)	383	115.1 (109.6-120.5)^{a}	59.4 (57.7-61.0)	40.6 (39.0-42.3)	2454 (2379-2529)^{b}	18.7 (18.1-19.3)	11.3 (10.8-11.8)	7.4 (7.0-7.8)
Obese (≥ 30.0)	312	116.2 (110.1-122.2)^{b}	60.7 (58.8-62.6)	39.3 (37.4-41.2)	2420 (2337-2503)^{c}	19.1 (18.4-19.8)	12.0 (11.3-12.6)	7.1 (6.7-7.5)
P		<0.0001	0.05	0.05	<0.0001	0.07	0.03	0.02
Education								
High school or less	284	117.7 (110.0-125.4)	62.8 (60.9-64.7)^{a}	37.2 (35.3-39.1)^{a}	2402 (2301-2502)	19.3 (18.5-20.1)	12.4 (11.6-13.1)	6.9 (6.4-7.4)^{a}
CEGEP	353	111.9 (106.8-116.9)	60.7 (58.9-62.5)^{a}	39.3 (37.5-41.1)^{a}	2374 (2301-2446)	18.8 (18.2-19.4)	11.7 (11.1-12.2)	7.1 (6.7-7.5)^{a}
University	510	118.9 (114.5-123.3)	57.0 (55.6-58.5)^{b}	43.0 (41.5-44.4)^{b}	2423 (2366-2479)	19.6 (19.1-20.1)	11.4 (11.0-11.9)	8.2 (7.8-8.5)^{b}
P		0.22	<0.0001	<0.0001	0.68	0.18	0.14	<0.0001
Reporting status								
Under reporters	185	69.0 (64.8-73.1)^{a}	55.1 (52.3-57.9)^{a}	44.9 (42.1-47.7)^{a}	1577 (1521.4-1633)^{a}	17.9 (16.9-18.9)	10.1 (9.3-11.0)^{a}	7.7 (7.1-8.4)
Plausible reporters	613	108.6 (105.5-111.7)^{b}	59.8 (58.4-61.1)^{b}	40.2 (38.9-41.6)^{b}	2248 (2218.0-2278)^{b}	19.3 (18.9-19.8)	11.9 (11.4-12.3)^{b}	7.5 (7.2-7.8)
Over reporters	348	155.5 (148.9-162.1)^{c}	61.6 (60.0-63.3)^{c}	38.4 (36.7-40.0)^{c}	3114 (3039.6-3188)^{c}	19.9 (19.3-20.6)	12.4 (11.9-13.0)^{b}	7.5 (7.1-8.0)
P		<0.0001	<0.0001	<0.0001	<0.0001	0.01	<0.0001	0.11

Daily intakes are presented as unadjusted means (95% Confidence Interval). *P* values are the main effect of sociodemographic characteristic on sugar intakes in the linear model. A *P* value <0.001 was considered statistically significant. ^{a,b,c} Subgroups means without a common superscript are different (Tukey-Kramer). ^{X} CEGEP is a preuniversity and technical college institution specific to the Province of Quebec.
